# A New Synopsis, or the Natural Order of Diseases: Containing Their Definition, Principles, and Treatment, with a New Pathology of Fever and Inflammation

**Published:** 1842-01

**Authors:** 


### ART. XII.

*A New Synopsis, or the Natural Order of Diseases: containing their Definition, Principles, and Treatment, with a New Pathology of Fever and Inflammation.* By ROBERT STEVENS, M.R.C.S. &c.—*London,* 1841. 8vo, pp. 175.

IN the introduction to this work, the author informs us that the principles of pathology and therapeutics are based on certain very simple laws, easily comprehensible to one who has a sufficient knowledge of anatomy and physiology; and at another place he affirms that "the first laws of practical medicine are as true and immutable as the rules of geometry, and are equally susceptible of demonstration." (p. 161.) Such being his opinion, he concedes less importance to the "details" and "incidental complications" of disease than is usual with practical writers. Yet, at p. 161, he says "the general rules of practical medicine, as far as they can be specified, are well known; but there are other points of far greater practical importance, which cannot be taught; they constitute the art or skill in medicine, and depend upon natural ingenuity solely." Now, we cannot exactly perceive on what the "natural ingenuity" is to be exercised, unless it be on the "details" and "incidental complications," seeing that the mastery of the general rules is a matter of *knowledge*, not of skill or ingenuity. Howsoever all this may be, we beg to say that if Mr. Stevens will indicate the immutable laws of medicine to which he alludes, we shall be exceedingly thankful, and he shall be unto us a "Magnus Apollo;" for we have always been accustomed to think that medicine is, of all sciences, the one least reducible to fixed laws.

Passing over other introductory matters, the first subject that presents itself is *the pathology of fever.* Our author makes some observations on the source of animal heat, and concludes in favour of the hypothesis which refers it to the chemical changes attendant on the conversion of arterial into venous blood, taking place in the capillaries during the process of assimilation throughout the body; he maintains further, that the circulation of the blood and the calorific process going on in the capillaries are under the control of the ganglial system of nerves. The opinion that animal heat has its origin in the changes which the blood undergoes in the capillary vessels is, perhaps on the whole, the most probable that has yet been advanced on the subject. With respect to the influence of the ganglial nerves on the calorific, or any other process carried on in the capillaries, we must repeat what we have said on a former occasion, that it is a point on which physiology calls for the aid of minute anatomy. It is quite futile to speculate on the influence of these nerves on the extreme blood-vessels, till we know whether they are actually distributed to them, which is hitherto ascertained only with reference to the arteries of particular parts of the body. On these data, which, we presume, he considers as certain as anything in geometry, Mr. Stevens founds a theory of fever. His exposition not being particularly lucid, we will not incur the risk of misinterpretation, but let him speak for himself.

"In fever the ganglial nervous power is augmented, as well as not only nutrition, but also the counterpart absorption and redigestion or decomposition of the old material; and as there is deficient appetite, the system is thus left to prey upon itself, the excitement called fever soon merging by exhaustion into a typhoid form. So fever exists not only where the vital actions are excited by the augmented circulation of good arterial blood, but also where the system has preyed upon itself, even where prostration and debility are induced. Hence the typhoid form, and though no definition can well explain both these opposite modifications of fever, yet their separate existence as different states of the same morbid action is perfectly intelligible. For when febrile action is continued, not only is there a quicker expending of the nutritious particles of the blood, but also the counterpart absorption of old material is equally augmented, the nutritious materials being so quickly worked and expended, that shortly there is no longer the proper stimulus for the nervous centres, namely, good arterial blood, and therefore the vitality is depressed, and the patient in a state of low typhus, the circulating fluids being attenuated and the general fibre relaxed, purpura supervening and a tendency to decomposition." (p. 26.)

It would be easy to state many objections to this theory, but there is one so obvious and conclusive that it alone may suffice. Admitting Mr. Stevens's view to apply in the case of common continued fever, where typhoid symptoms occur only in the advanced stage, it affords no explanation of those severer forms of fever in which the circulation is depressed and the surface cool from the commencement; yet these are the very cases to which a true theory of fever ought to apply most exactly; because they exhibit the disease in its most essential form, as produced by the most concentrated operation of its causes. We fear, therefore, that Mr. Stevens's theory must share the fate of various similar products of human ingenuity.

We next encounter *the pathology of inflammation*, of which our author's view is briefly this. Inflammation is an affection of a part resembling that which obtains throughout the system in fever, in as far as there is, in both cases, augmented circulation and heat: but in fever, the assimilation is no otherwise altered than in the increased rate of action, deposition and absorption going on more rapidly than in health; while, in inflammation, we have not only augmented circulation and heat in the part affected, but also anormal deposits, as coagulating lymph, pus, &c., with a new action of the absorbents. There is doubtless truth, though no novelty, in the notion that the phenomena of inflammation may be greatly affected by the mechanical and other influences of its own products. We do not think, however, that our author is very happy in his illustrations of the modus operandi of such causes. The following, we should say, was precisely in the worst style of Magendie. Mr. Stevens observes that "if excitement take place from irritation or other causes, we have an increase of secretion; but if obstruction take place from excitement, or any cause such as the error loci of the corpuscular pathologists, we have the ulcerative inflammation, and effusion of lymph," &c. (p. 43.) He adds, in a note, "The following simple experiment may illustrate this obstruction from excitement: If a quantity of sand, not too fine or dry, be put into a glass or other funnel, it will gradually fall through the pipe, unless pressure be made on the upper surface of the sand, when it will cease to fall through, the particles becoming packed at the bottom of the funnel." Is this written gravely, or is it a quiz on the "phénomènes physiques de la vie?"

We must now notice the *nosology*, although we have elsewhere expressed our belief that, whatever may be the utility of detached nosological groups, all attempts at a general systematic arrangement of diseases should be abandoned, as tending to connect them only by relations which are false or useless. We speak of course with reference to the actual state of knowledge, and pretend not to determine what rank nosology may hereafter hold among the medical sciences.

Mr. Stevens arranges all diseases under the two primary divisions of *sthenic* and *asthenic*. He maintains that there is not a single genus in Cullen's second and fourth classes (*neuroses* and *locales*) that might not be transferred into his first and third (*pyrexiæ* and *cachexiæ*), or into the *sthenic* and *asthenic* divisions of Mr. Stevens's system.

This has accordingly been done in the nosology before us, Cullen's classes being reduced from four to two, and his orders from twenty to eighteen. The orders of Mr. Stevens's system are nine in each class, and are arranged in contrasting pairs, each genus of sthenic disease having its asthenic analogue. The classes and orders, then, stand thus:

| CLASS I. STHENICÆ. | | CLASS II. ASTHENICÆ. | |
|---|---|---|---|
| Order 1. | Febres. | Order 1. | Marcores. |
| 2. | Phlegmasiæ. | 2. | Adynamiæ. |
| 3. | Eruptiones. | 3. | Cachexiæ. |
| 4. | Impetigines. | 4. | Parasiticæ. |
| 5. | Tumores. | 5. | Ectopiæ. |
| 6. | Hæmorrhagiæ. | 6. | Profluviæ. |
| 7. | Spasmi. | 7. | Dyscinesiæ. |
| 8. | Dyosorexiæ. | 8. | Dysæsthesiæ. |
| 9. | Vesaniæ. | 9. | Dementiæ. |

It must be admitted that Cullen's class of *locales* is very badly constructed, and Mr. Stevens has, we think, done well in rejecting it; but we do not see how any nosology can get on without a class of *neuroses*, for purely nervous diseases appear to be associated more naturally, and on plainer grounds than any others, inasmuch as they manifest their principal effects on one system only, while other diseases more or less implicate all the systems. For example, *idopathic epilepsy* is considered by every one as a nervous disease, there is no dispute about it; but *typhus* which implicates every system and function, is considered by some as a nervous disease, by others as an inflammatory disease, and by others as a disease of the blood.

We do not think we should be occupying our time profitably to the reader in analysing Mr. Stevens's nosology. In common with all others, several of its genera hold certain places for reasons doubtless satisfactory to the author, but inscrutable to every one else. For example, Cullen places *fractura* and *tinea* under the same order, *dyalyses :* a broken thigh and a scald-head have certainly a wonderful affinity, but surely not more than *icterus* and *necrosis*, which Mr. Stevens has brought together under the order *adynamiæ*.

If Mr. Stevens has failed in framing a nosology, it must be allowed that nobody has succeeded much better; but there is one thing, unconnected entirely with the subject of classification, which we cannot let pass. No one should think of publishing a nosology who does not possess a sufficient knowledge of the classical languages from which the terms of the science are adopted or derived.

If Mr. Stevens had possessed such knowledge, he never could have latinized καταῤῥυς by *catarrha*, nor βουλιμια by *bulimea*, nor φθισις by *phthysis;* nor could he have designated *toothach* by such a term as *ondontalgitis*, which would signify "inflammation of a toothach." These are not errors of the press, for they are reiterated in different parts of the book.

Mr. Stevens has attempted a sort of compendium of treatment, in conjunction with the definitions of diseases. We do not conceive that such an object could be very successfully accomplished, but the thing might certainly be better done than it is here. Look, for example, at the treatment of *erysipelas :*

"Reduce the inflammation on general principles; by the use of saline purgatives and the emetic tartar; by evaporating lotions; by cutaneous incisions, which empty the vessels and relieve the tension." (p. 54.)

Suppose that a student, taking Mr. Stevens's book for his guide, were to give emetic tartar in a case of typhoid erysipelas, what would be the probable result?

There is an appendix to the nosology treating of "symptomatic affections;" and an appendix to the whole book "on medical practice and medical science." These we have not space to notice, and, were it otherwise, the less perhaps that was said about them the better. From Mr. Stevens's affirmation that "really scientific men are successful in almost all cases submitted to their care, perhaps only excepting cases of phthysis and malignant diseases of structure," we are led to infer that he must be a wonderfully successful practitioner; in which case we would recommend him to lay aside the pen, for authorship is not his *forte*.

---

